# Small Cell Lung Cancer in the Course of Idiopathic Pulmonary Fibrosis—Case Report and Literature Review

**DOI:** 10.3390/curroncol29070401

**Published:** 2022-07-18

**Authors:** Maria Grodkiewicz, Pawel Koziel, Izabela Chmielewska, Marta Adamczyk Korbel, Janusz Milanowski

**Affiliations:** 1 Students Association at the Department of Pneumonology, Oncology and Allergology, Medical University of Lublin, 20-950 Lublin, Poland; marysiagrodkiewicz@gmail.com (M.G.); pawelkoziel61@gmail.com (P.K.); 2 Department of Pneumonology, Oncology and Allergology, Medical University of Lublin, 20-950 Lublin, Poland; jmilanowski@op.pl; 3 Independent Public Clinical Hospital No. 4 in Lublin, 20-950 Lublin, Poland; mako99@o2.pl

**Keywords:** idiopathic pulmonary fibrosis (IPF), lung cancer (LC), small cell lung cancer (SCLC), carboplatin–etoposide chemotherapy, pirfenidone

## Abstract

Idiopathic pulmonary fibrosis is a poorly prognosed form of progressive interstitial pneumonia. Patients with IPF have a significantly increased risk of developing lung cancer, which further worsens the course of the disease. The most common histological types of LC among patients with IPF are squamous cell carcinoma and adenocarcinoma. Furthermore, all LC treatment modalities can lead to developing an acute IPF exacerbation. In this report, we present a rare case of coexistence of IPF and small cell lung cancer in a 76-year-old patient with chronic obstructive pulmonary disease, and a former smoker. For over 2 years, the patient was treated with an anti-fibrotic drug-pirfenidone, which slowed down the progression of IPF. Unfortunately, after being diagnosed with an active SCLC, the patient was excluded from further participation in the pirfenidone drug program. SCLC is characterized by high aggressiveness, rapid growth and high metastatic potential; therefore, it is necessary to apply antitumor treatment as soon as possible. The described patient was treated with carboplatin–etoposide chemotherapy. Early treatment tolerance was good and after two cycles of cytotoxic treatment, a partial response was present in CT. The presented case emphasizes the need for further research to determine the treatment regimens in patients with coexisting IPF and LC and the appropriateness of antifibrotic treatment in them. In addition, it can help to choose the treatment method for similar patients, indicating a combination of carboplatin and etoposide as an effective and, at the same time, relatively safes method in terms of the risk of IPF’s exacerbation.

## 1. Introduction

Idiopathic pulmonary fibrosis (IPF) is a specific form of chronic, progressive, fibrosing interstitial pneumonia of unknown cause. It is a rare disease with an estimated prevalence ranging from 10–60 cases per 100,000 people, which occurs primarily in older adults. IPF is defined by the histopathologic and/or radiologic pattern of usual interstitial pneumonia (UIP). It leads to a progressive worsening of dyspnea and an irreversible decline of lung function. The clinical course of IPF varies and is difficult to predict, nevertheless, the prognosis is poor and the mean survival is 3–5 years from diagnosis. However, there are two anti-fibrotic drugs—pirfenidone and nintedani—which decrease physiological progression and likely improve progression-free survival [1,2].

There are several pulmonary and extrapulmonary comorbid conditions that affect the course and prognosis of IPF, such as chronic obstructive pulmonary disease, pulmonary hypertension, congestive heart failure and lung cancer (LC). It is worth noting that among them, lung cancer has the biggest impact on mortality in the setting of IPF, and LC survival is significantly worse than in patients with IPF without LC (median survival 38.7 months vs. 63.9 months) [3]. The relative risk of developing LC in IPF is approximately seven times higher than in the general population [3], and the prevalence of LC in patients with IPF is reported to be 3–48% [4]. The most frequent histologic type of LC in IPF’s course is squamous cell carcinoma, followed by adenocarcinoma, and small cell lung cancer (SCLC) comprises approximately 15–20% of the cases, which is similar to its incidence in the general population [3,5]. 

SCLC is an aggressive cancer of neuroendocrine origin, which is strongly associated with cigarette smoking. It is characterized by rapid doubling time and early dissemination in lymph nodes and distant organs. Despite its high sensitivity to chemotherapy and radiotherapy, survival rate is low due to its high relapse rate. Considering the fact that the prognosis is poor even with IPF alone, when IPF coexists with SCLC, the patient has a very low chance of survival [6].

The aim of this study is to present a rare case of coexistence of IPF and SCLC and to analyze the available therapeutic options, which are very limited due to the fact that in patients with IPF, all LC treatment modalities can lead to lung function deterioration [7].

## 2. Case Report

**Figure 1:**
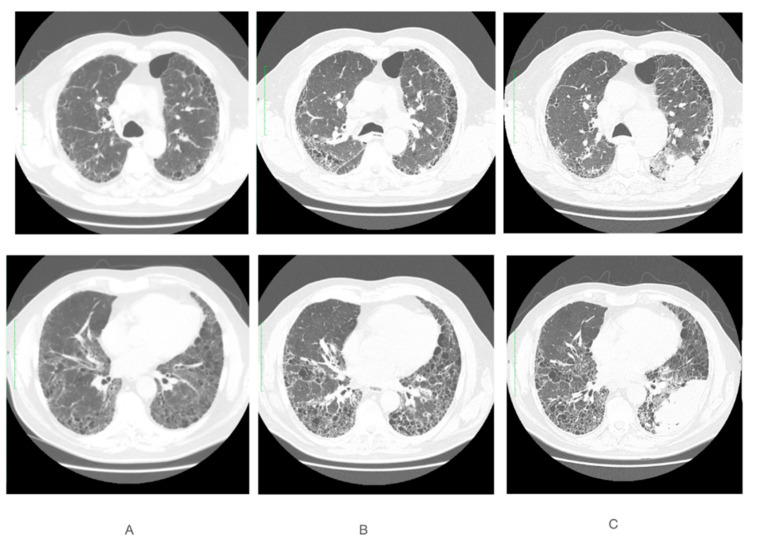
CT scans performed at two depths (upper and lower views) at different time points: (**A**) November 2019, (**B**) March 2021 and (**C**) February 2022.

A 76-year-old man was admitted to the Department of Pneumonology, Oncology and Allergology in November 2019 due to symptoms that had been intensifying for several months: dry cough, exercise dyspnea, exercise intolerance and general weakness. He had been suffering from chronic obstructive pulmonary disease since 2013; for this reason, he was being treated with home oxygen therapy. The patient is a former smoker of 40 pack-years. His past medical history includes arterial hypertension and ischemic heart disease. The computer tomography examination revealed the features of massive honeycomb pulmonary fibrosis, with a predominance distribution in subpleural and basal areas. Moreover, reticular abnormalities such as thickening of intra-and interlobular septa, increased lung density, bronchial tree dilatation and the presence of emphysema were visualized, and in the HRCT option, small air traps during the expiratory phase (Figure 1A). In pulmonary function tests, restrictive-type ventilation disorders were present: decreased FEV1 (forced expiratory volume in 1 s) 66% of predicted values (% pred.) and decreased VC MAX (vital capacity) 64% pred. with normal FEV1%/VC MAX ratio, significantly decreased TLC (total lung capacity) 61% pred. and decreased DLCO (diffusing capacity for carbon monoxide) 37% pred. Infection of Mycobacterium tuberculosis was excluded. No skin or mucosal changes, arthritis or muscle weakness were present. Laboratory tests revealed no cytopenia, ANA (antinuclear antibodies) and ANCA (antibodies against the cytoplasm of neutrophils) were absent, which is normal. On this basis, a systemic cause of changes in the lungs was excluded. Due to the fact that in the upper parts of the lungs, changes in the form of a milky glass were also found in CT, and in bronchoalveolar lavage (BAL) there was an increased percentage of lymphocytes, treatment with oral glucocorticosteroids was initiated, which after two weeks of treatment, due to the lack of effectiveness, was reduced and finally discontinued. Based on the performed radiological diagnostics, known causes of pulmonary fibrosis were excluded and idiopathic pulmonary fibrosis was diagnosed. Due to clear radiological presentation of the disease and underlying chronic obstructive respiratory disease, histopathological confirmation was not performed. The patient was qualified for antifibrotic IPF treatment. Pirfenidone treatment was implemented in December 2019. After 11 months of pirfenidone treatment, an increase in TLC 80% pred. and DLCO 55% pred. values were noted. In March 2021, the patient had a follow-up CT of the chest with the HRCT option, which showed a slight progression of pulmonary fibrosis with a honeycomb image (Figure 1B). In February 2022, he was re-hospitalized at the clinic for the diagnosis of a large infiltrative lesion on the chest CT, including the lower and partially upper lobe of the left lung and lymphadenopathy of both cavities (Figure 1C), which were not present on the CT from March 2021. Before hospitalization, he reported dyspnea at rest for about two weeks and increased cough with residual secretions in the respiratory tract. He denied hemoptysis and chest pain. Oxygen saturation was 89% without oxygen therapy. The results of arterial blood gases were as follows: pH 7.43, pCO_2_ 44 mmHg, and pO_2_ 48 mmHg. During hospitalization, a positive COVID-19 antigen test result was obtained (patient was not vaccinated against COVID-19). 

**Figure 2:**
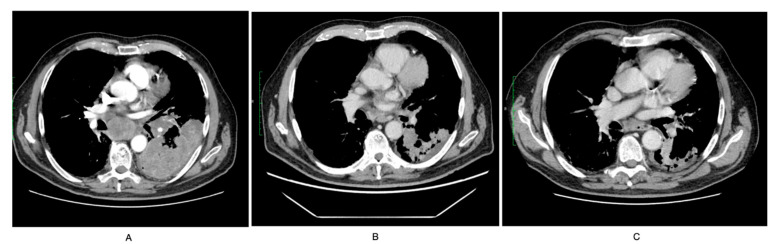
Efficacy of chemotherapy: before treatment (**A**), after 2 cycles (**B**) and after 4 cycles (**C**).

In a chest CT performed in March 2022, polycyclic confluent tissue masses were visualized, infiltrative in the left lung occupying a significant part of the lower lobe and the dorsal parts of the upper lobe with radiological features of the neoplastic lesion. Compared to the CT performed in February, progression of nodal lesions in the mediastinum and hilars was visible (Figure 2A). Nodal changes modeled adjacent structures, including vessels. In addition, significant narrowing of the low lobe artery with the possibility of infiltration, progression of fluid in the left pleural cavity and in the pericardium. In the histopathological examination of the material from endobronchial ultrasound bronchoscopy (EBUS), small cell lung cancer was diagnosed. Immunohistochemical reactions were performed: synaptophysin+, CD56+, Ki67+ in 70% of neoplastic cells. The diagnosis of T4N2M0 small cell lung cancer was confirmed. The presence of an active neoplastic disease is a criterion that excludes participation in the pirfenidone drug program; the patient had to discontinue antifibrotic treatment. Systemic treatment of SCLC was started. Patient was qualified to receive a carboplatin–etoposide regimen, and early treatment tolerance was good. After two cycles of cytotoxic treatment, a partial response was present on theCT (Figure 2B), which was persistent after four cycles (Figure 2C). Cytotoxic treatment was well tolerated, and 4 months PFS was reached. Since the tumor regression was present and there was no longer a risk of bleeding due to central tumor invasion, antifibrotic and antiangiogenic nintedanib treatment was implemented. 

## 3. Discussion

Lung cancer has been shown to significantly worsen the course of the disease and the prognosis of patients with IPF. Despite many studies on the cause of frequent LC development in the course of IPF, the underlying pathomechanism has not yet been clearly confirmed [8]. Proven risk factors for lung cancer in IPF are smoking, old age, male gender and emphysema [9]. Several possible pathogenesis mechanisms linking LC and IPF are suspected. The pathogenesis of both of these diseases is characterized by epigenetic and genetic change alterations, abnormal expression of microRNAs (miRNAs), cellular and molecular aberrances, such as an altered response to regulatory signals, delayed apoptosis or cell-to-cell communication, along with activation of specific signaling transduction pathways [10,11]. Moreover, the genetic basis of idiopathic interstitial pneumonia and lung cancer as mutations in the surfactant proteins A1 or A2 has been proven [12,13,14].

The guidelines for the treatment of lung cancer for the general population are unambiguous, but there are still no commonly accepted recommendations for the group of patients with IPF, especially for the rare co-occurrence of IPF with SCLC. Various treatment methods for lung cancer, including surgical intervention, chemotherapy and radiation therapy, carry the risk of inducing an acute exacerbation of IPF, which can be fatal [3,15]. In one of the very few cases of coexistence of IPF and SCLC that we were able to find, the patient expired due to respiratory failure caused by an acute exacerbation of IPF 3 months after the implementation of SCLC treatment-chemotherapy with irinotecan and carboplatin [16]. Therefore, finding a safe and effective method of treatment for these patients is challenging.

Recent studies show that pirfenidone combined with carboplatin-based regimens or immune checkpoint inhibitors might be a safe first-line chemotherapy for patients with IPF and non-small cell lung cancer (NSCLC), which accounts for the vast majority of LC in the course of IPF [17]. SCLC occurs less frequently (20.48% of LC in patients with IPF), but it has a more aggressive course and a shorter life expectancy [18,19,20]. Withdrawal from SCLC treatment is associated with rapid tumor growth, metastasis and poor prognosis. Due to the high sensitivity of lung cancer to chemotherapy, several studies have been conducted on the efficacy and safety of treatment with platinum agents plus etoposide as first-line chemotherapy for SCLC with pre-existing interstitial lung disease (ILD), including IPF [21,22,23]. Yoshida et al. reported that during this treatment, only one (2%) out of the 52 patients developed an acute exacerbation of ILD, the overall response rate was 69%, the median progression-free survival period was 4.5 months and the median survival time was 9.4 months [21]. 

Nintedanib, another antifibrotic agent, is frequently used in combination with docetaxel for treatment of NSCLC [24]. Nintedanib is a triple-action angiokinase inhibitor that blocks the activity of kinases of vascular endothelial growth factor receptors (VEGFR 1–3), platelet-derived growth factor receptors (PDGFR α and β) and fibroblast growth factor receptors (FGFR 1–3) [25]. However, there are limited data exploring the effectiveness of nintedanib monotherapy against NSCLC in patients with coexistence of IPF. Cases in which nintedanib as a single agent prevented the progression of IPF and the associated squamous cell carcinoma or adenocarcinoma simultaneously have been reported in the literature [26,27]. Kai et al. described a case in which, after seven months of NSCLC treatment with nintedanib alone, the primary tumor, pleural dissemination and mediastinal lymph node metastasis regressed and at the same time, IPF progression did not occur [28]. Moreover, a case described by Shiratori et al. proved that nintedanib monotherapy might be a therapeutic option for NSCLC patients with IPF who are unable to tolerate chemotherapy [29]. Nevertheless, it still remains uncertain whether nintedanib can reduce LC incidence in patients with IPF, prolong the survival time of patients with IPF-associated LC or reduce acute exacerbation of IPF associated with chemotherapy. However, nintedanib is not used in the treatment of SCLC. The main contradiction to use it in this setting is centrally localized tumor with the risk of invasion on main arteries. That is the reason why it was not implemented as IPF simultaneous treatment. The treatment with nintedanib was started in further course of treatment since there was a rapid shrinkage of tumor present. 

For over 30 years, platinum-based chemotherapy has remained the primary therapeutic option for patients with SCLC. The recent CASPIAN and IMpower133 studies on the effectiveness of the combination of chemotherapy and immunotherapy in this group of patients have opened a completely new chapter in SCLC treatment. They proved that the addition of an antibody blocking the PD-L1 molecule (atezolizumab in IMpower133, durvalumab in CASPIAN) to first-line chemotherapy (based on platinum and etoposide) has a more favorable effect on patients’ survival than chemotherapy alone. In both studies, the median overall survival of previously untreated patients with extensive stage SCLC was significantly longer in the group receiving immunotherapy in combination with chemotherapy compared to the group receiving chemotherapy alone (CASPIAN: 13 vs. 10.3 months; IMpower133: 12.3 vs. 10.3 months). The safety profile was acceptable. These two immune checkpoint inhibitors proved to be the first agents in the last decades to determine an improvement in outcomes of patients with extensive stage SCLC patients, so it led to a new standard of care [30]. The obtained results constitute the basis for a change in clinical practice, especially in highly developed countries. It should be noted that the improvement in survival rates associated with the addition of atezolizumab or durvalumab to chemotherapy it is not as spectacular as in NSCLC. However, the results of these studies show that SCLC patients may benefit from immunotherapy, which is the first real progress in the prognosis of SCLC patients in many years. Ide et al. were the first to report a case of an NSCLC patient with IPF who experienced a durable response to nivolumab for >1 year without exacerbation of IPF [31]. Unfortunately, it has also been described that immunotherapy (firstly with pembrolizumab and then atezolizumab) has induced pneumonitis in a case of a patient with squamous cell lung cancer and IPF [32]. The presence of baseline lung disease has been shown in several studies to increase the risk of pneumonitis. In patients with poor lung function, it may lead to a worse prognosis. Taking in to consideration the high risk of respiratory failure in case of immune-related pneumonitis, we did not include combination treatment. However, there are not any reports on the use of immunotherapy in patients with SCLC and IPF. Hence, there is a need to continue research on the role and safety of immunotherapy in various groups of patients with SCLC and to find the most optimal chemotherapy and immunotherapy regimen.

Since the coexistence of both diseases is rare, so far not enough clinical trials have been conducted to determine the optimal chemotherapy regimen for patients with IPF and SCLC that would not adversely affect the course of IPF. Therefore, when choosing a chemotherapy method, we can only use similar cases described in the literature. Among these few cases are two SCLC and IPF patients with poor lung function described by Zhang et al. They also received chemotherapy based on a derivative of platinum and etoposide. At the same time, anti-fibrotic treatment was continued. After treatment, both patients’ clinical condition and lung function improved [33].

In conclusion, an individualized approach is essential when choosing a treatment method for patients with IPF and SCLC. Chemotherapy seems to be the most effective method against SCLC, and at the same time, relatively the safest method in terms of the risk of exacerbation of IPF. The case of the patient described by us emphasizes the need for research into the development of new therapeutic agents that would enable effective treatment of both SCLC and IPF.

## Data Availability

The data presented in this study are available on request from the corresponding author.
